# Receptividade à vacina contra o papilomavírus humano: uma revisão sistemática

**DOI:** 10.26633/RPSP.2019.22

**Published:** 2019-02-06

**Authors:** Lídia Ester Lopes da Silva, Maria Liz Cunha de Oliveira, Dayani Galato

**Affiliations:** 1 Programa de Pós-Graduação em Ciências e Tecnologia da Saúde (PPGCTS) PPGCTS Faculdade de Ceilândia (FCE) Universidade de Brasília (UnB) BrasíliaDF Brasil Universidade de Brasília (UnB), Faculdade de Ceilândia (FCE), Programa de Pós-Graduação em Ciências e Tecnologia da Saúde (PPGCTS), Brasília (DF), Brasil.; 2 Programa de Mestrado Profissional em Ciências da Saúde Programa de Mestrado Profissional em Ciências da Saúde Escola Superior de Ciências da Saúde (ESCS) BrasíliaDF Brasil Escola Superior de Ciências da Saúde (ESCS), Programa de Mestrado Profissional em Ciências da Saúde, Brasília (DF), Brasil.

**Keywords:** Vacina, papilomavírus humano, aceitação pelo paciente de cuidados de saúde, cooperação do paciente, Vaccines, papillomaviridae, patient acceptance of health care, patient compliance, Vacunas, papillomaviridae, aceptación de la atención de salud, cooperación del paciente

## Abstract

**Objetivo.:**

Caracterizar a receptividade à vacina contra o papilomavírus humano (HPV) e descrever as barreiras e os facilitadores dessa receptividade.

**Métodos.:**

Trata-se de uma revisão sistemática conforme o protocolo PRISMA 2015. Os repositórios MEDLINE e *Web of Science* foram consultados utilizando combinações dos termos *papillomavirus*, *vaccine*, *adherence* e *acceptance* para identificar artigos publicados de 2006 a 2017. Foram incluídos artigos originais em qualquer idioma e excluídos artigos duplicados. Foram analisadas identificação do artigo, tipificação metodológica e caracterização da amostra. A receptividade foi caracterizada em termos de aceitação e adesão.

**Resultados.:**

Foram identificados 212 artigos, sendo 10 selecionados para análise. A maioria dos estudos evidenciou receptividade favorável, porém heterogênea, havendo maior aceitação do que adesão, principalmente por adolescentes do sexo feminino. Foram identificados 11 facilitadores e nove barreiras à receptividade, com destaque para conhecimento relativo ao tema e padrão de comportamento individual frente ao problema. Observou-se a inexistência de um método padronizado que avalie a temática e a imprecisão dos conceitos associados a aceitação e adesão. Diante disso, o estudo propôs conceitos de aceitação (intenção voluntária de receber uma vacina ou concordar que a mesma representa uma boa estratégia preventiva) e adesão (ato de iniciar a vacinação e completar o esquema).

**Conclusões.:**

Novos estudos são necessários para aprofundar a análise dos preditores da receptividade. Sugere-se a construção de um instrumento baseado na percepção do público alvo e em conceitos precisos de aceitação e adesão, que possibilite melhor compreensão do fenômeno e estimule a adesão e o alcance de coberturas vacinais adequadas.

## Abstract (en)

**Objective.:**

To characterize the receptivity to human papillomavirus (HPV) vaccine and to describe factors that hinder or facilitate receptivity.

**Method.:**

A systematic review was performed based on the 2015 PRISMA protocol. MEDLINE and Web of Science were searched using combinations of the terms papillomavirus, vaccine, adherence, and acceptance to identify articles published from 2006 to 2017. Original articles published in any language were included, and duplicate articles were excluded. Information was collected regarding article identification, methodological design, sample characteristics, and description of contents. Receptivity was characterized in terms of acceptance and adherence.

**Results.:**

Of 212 articles identified, 10 were selected for analysis. Most showed favorable, but heterogeneous, receptivity, with more acceptance than adherence, especially on the part of female adolescents. The analysis identified 11 facilitators and nine barriers to receptivity, especially knowledge of the theme and individual pattern of behavior regarding the problem. The analysis also detected the absence of a standardized method to evaluate receptivity and the imprecision of the concepts associated with acceptance and adherence. Therefore, a definition was proposed for acceptance (voluntary intention of receiving the vaccine or agreement that the vaccine is a good prevention strategy) and adherence (initiation and completion of the vaccination scheme).

**Conclusions.:**

Additional studies are required to further investigate predictors of receptivity. For that, the development of an instrument based on the perceptions of target publics and employing precise concepts of acceptance and adherence is recommended, so as to allow a better understanding of the phenomenon and stimulate adherence and the achievement of adequate vaccine coverage.

## RESUMEN

**Objetivo.:**

Caracterizar la receptividad con respecto a la vacuna contra el virus del papiloma humano (VPH) y describir los factores inhibidores o facilitadores correspondientes.

**Métodos.:**

Se trata de una revisión sistemática según el protocolo PRISMA 2015. Se consultaron las bases de datos MEDLINE y Web of Science, utilizando combinaciones de los términos “papilomavirus”, “vaccine” [vacuna], “adherence” [adhesión] y “acceptance” [aceptación] para encontrar artículos publicados desde el 2006 hasta el 2017. Se incluyeron artículos originales en cualquier idioma y se excluyeron los artículos duplicados. Se analizaron la identificación del artículo, la tipificación metodológica y las características de la muestra. La receptividad se caracterizó en términos de aceptación y adhesión.

**Resultados.:**

Se encontraron 212 artículos de los cuales se seleccionaron 10 para el análisis. En la mayoría de los estudios se demostró una receptividad favorable, pero heterogénea, con mayor aceptación que adhesión, principalmente por parte de adolescentes del sexo femenino. Se identificaron once factores facilitadores y nueve inhibidores de la receptividad, entre los cuales se destacan el conocimiento del tema y el patrón de comportamiento individual frente al problema. Se observó que faltaba un método normalizado que permitiera evaluar la receptividad y la imprecisión de los conceptos relacionados con la aceptación y la adhesión. Por lo tanto, se propuso una definición de aceptación (intención voluntaria de recibir una vacuna o expresión de acuerdo de que la vacuna representa una buena estrategia preventiva) y adhesión (acto de iniciar y finalizar el esquema de vacunación).

**Conclusiones.:**

Se necesitan nuevos estudios para profundizar el análisis de los factores de predicción de la receptividad. Para ello, se sugiere la creación de un instrumento basado en la percepción del público destinatario y el empleo de conceptos precisos de aceptación y adhesión, con el fin poder comprender mejor el fenómeno y fomentar la adhesión y el logro de tasas adecuadas de cobertura con la vacuna.

O papilomavírus humano (HPV) apresenta elevado potencial oncogênico para causar lesões na mucosa genital (1). Nesse contexto, a vacina contra o HPV (vcHPV) é uma estratégia preventiva (2) contra afecções como verrugas e neoplasias (1) e, especialmente, contra o câncer cervical (3) – o quarto câncer mais comum entre as mulheres no mundo, afetando principalmente os países menos desenvolvidos (2, 4-6).

Dois tipos de vacinas foram desenvolvidos a fim de prevenir a disseminação do HPV (6): a bivalente, que protege contra os subtipos 16 e 18 (6), e a quadrivalente, que protege contra os vírus 6, 11, 16 e 18 (1, 7-10). Em 2006, o uso da forma quadrivalente foi autorizado nos Estados Unidos (1, 9) e passou, então, a ser comercializada em outras partes do mundo (10). A literatura mostrou bons resultados associados à vacina, com elevada segurança e eficácia (em torno de 80%) na prevenção de neoplasia cervical e lesões genitais em mulheres (7) e redução de 56% na incidência da infecção em adolescentes americanos (11).

A vcHPV é utilizada como intervenção preventiva em diversos países, como França (12, 13) e Suécia (14). Embora a vacina possa ser administrada em diferentes idades, o foco é o público infantil e adolescente em virtude dos melhores resultados da vacinação em idades precoces (7), contexto no qual a imunização gratuita em escolas (3) tem garantido boas coberturas vacinais (1, 2, 10). No Brasil, o Ministério da Saúde incorporou o imunobiológico em 2014 (3), com inserção da vacina no Calendário de Vacinação do Adolescente. O atual esquema prevê duas doses aplicadas com intervalo de 6 meses (15).

Apesar do alto potencial preventivo (10), a vcHPV tem gerado controvérsias (3) que repercutem na aceitação (3) e na adesão (16) por parte dos usuários. Apontam-se diferenças na receptividade entre países: No Reino Unido, a receptividade foi alta (2, 17) chegando a 86,7% entre as adolescentes (10). Já nos Estados Unidos, a receptividade foi de 37% (18), taxa considerada baixa por pesquisadores da área (2, 7, 15); e a receptividade foi muito baixa no Japão, onde ocorreu uma rejeição à vacina (3). Desse modo, a baixa receptividade reportada em algumas nações tornou-se um desafio para o alcance de metas de cobertura vacinal (3), principalmente no que tange à identificação de fatores associados à não adesão.

Considera-se “receptividade” uma palavra alusiva a receber (19) a vacina em termos de “aceitação” e de “adesão”. Contudo, observam-se estudos que discorrem sobre o fenômeno sem oferecer uma definição precisa desses termos (14, 16), havendo ainda o uso de termos que, embora possuam significados literais diversos, no contexto relativo à imunização assumem o sinônimo de aceitação e adesão, como aceitabilidade (20), aderência (21) e decisão de vacinar (22). A diversidade de termos e a falta de concordância sobre uma definição comum é uma limitação da literatura científica, que não diminui a importância de avaliar aspectos referentes à imunização para além da cobertura em si (23).

Diante do surgimento de controvérsias relacionadas à receptividade à vcHPV e do desafio de entender os conceitos “adesão” e “aceitação”, tornou-se relevante mapear a literatura científica quanto ao objeto receptividade. Nessa vertente, analisar estudos focados na perspectiva do grupo-alvo e no levantamento de variáveis elencadas proporciona um olhar panorâmico do fenômeno (10) com vistas a desenvolver estratégias de proteção à saúde de forma adequada à realidade do público–alvo. Assim, o presente estudo teve como objetivo caracterizar a receptividade à vcHPV, por meio da aceitação e da adesão, bem como descrever os fatores relacionados a essa receptividade.

## MATERIAIS E MÉTODOS

Realizou-se uma revisão sistemática, com abordagem descritiva, conforme o protocolo *Preferred Reporting Items for Systematic Reviews and Meta-Analyses* (PRISMA) versão 2015 (24). A pergunta norteadora foi: “como a literatura científica evidencia a aceitação e a adesão à vcHPV por crianças e/ou adolescentes?” Inicialmente, definiram-se termos de busca (*medical subject headings*, MeSH), organizados conforme a seguinte estratégia: “TI (papillomavirus AND vaccine* OR Papillomavirus Vaccines OR Papillomaviridae AND vaccine* OR HPV AND vaccine*) AND TI (Medication Adherence OR adherence OR Patient Compliance OR accept*) AND (child OR adolescent) NOT review”. Foi dada preferência para os termos “vacina”, “papilomavírus”, “aceitação” e “adesão” no título (designado como *TI*), com os demais termos no corpo dos artigos.

As bases de dados escolhidas foram a MEDLINE e a *Web of Science,* com acesso pelo Portal de Periódicos da Coordenação de Aperfeiçoamento de Pessoal de Nível Superior (CAPES). Após rodar a estratégia de busca, foram aplicados os critérios de inclusão e exclusão, sendo incluídos artigos originais que abordassem a aceitação e a adesão à vcHPV, publicados em qualquer idioma, e que avaliassem crianças e/ou adolescentes; e excluídos artigos publicados antes de 2006 (ano de início da comercialização da vacina) (2, 9) e textos duplicados, ­realizando-se, subsequentemente, a seleção por leitura dos títulos e resumos.

O uso de determinadas palavras-chave, como “papillomavirus”, “vaccine”, “medication adherence”, “patient compliance” e “accept”, também gera como resultado da busca artigos que discorrem sobre questões bioquímicas relacionadas ao vírus; pesquisas com vacinas ainda em testes; estudos com adultos ou animais; protocolos de intervenções para aumentar a aceitação à vacina; pesquisas com responsáveis de adolescentes; estudos com grupos específicos, como homossexuais e universitários; textos como revisões sistemáticas, ensaios teóricos e artigos não originais, dentre outros. Assim, após a leitura refinada dos títulos e resumos, foi possível verificar quais artigos realmente seriam fontes de dados, conforme o protocolo estabelecido.

Já a etapa de leitura integral dos textos ocorreu de modo independente por dois revisores em locais e momentos distintos para confirmar quais textos seriam incluídos na revisão. Depois da seleção dos textos, extraíram-se informações de identificação do artigo, tipificação metodológica, caracterização da amostra e descrição do conteúdo.

Em relação à identificação do artigo, foram registrados os nomes do pesquisador principal e dos colaboradores, o ano de publicação e o país de origem (local de realização do estudo, não havendo restrições geográficas quanto à origem). A tipificação metodológica enfocou o desenho do estudo, o nível de evidência (classificação dos estudos, conforme o desenho metodológico, nas categorias 1a, 1b, 1c, 2a, 2b, 2c, 3a, 3b, 4 e 5, sendo 1a a categoria de maior rigor metodológico e 5 a de menor confiabilidade) (25), técnica de coleta de dados e detalhes da análise estatística, caso houvesse.

Para a caracterização da amostra, foram levantadas informações sobre os sujeitos da pesquisa. Apesar do foco em crianças ou adolescentes, também foram incluídos artigos envolvendo sujeitos que tivessem participado das pesquisas concomitantemente ao público-alvo, como pais ou responsáveis. Foram coletadas ainda informações sobre sexo (masculino ou feminino) e idade (0 a 9 anos e 10 a 19 anos, intervalos compreendidos respectivamente como infância e adolescência pela Organização Mundial da Saúde) (26). Artigos que incluíram participantes com idades próximas ao extremo foram mantidos para evitar a perda de informações relativas aos participantes. Foram ainda coletadas informações sobre o estado vacinal (vacinado ou não vacinado) e sobre o total de participantes envolvidos em cada pesquisa.

Quanto à descrição do conteúdo, foram levantadas informações sobre definições de aceitação e adesão e conceitos referidos pelos autores, caso tenham sido explanados; forma de avaliação, ou seja, descrição do modo pelo qual os autores avaliaram o grau de receptividade reportado por suas respectivas amostras; grau de receptividade reportado pelo estudo (mensuração do quanto os participantes aceitaram ou aderiram à vacina); e fatores relacionados à receptividade, com identificação de preditores que atuam como barreiras ou facilitadores à aceitação e à adesão. Esses determinantes foram identificados nos trabalhos caso fossem citados ao menos uma vez, sem levar em consideração a frequência de repetição.

Para a organização das informações adotou-se o programa Microsoft Excel 2016. Assinalou-se a correspondência e a frequência absoluta de cada variável concernente a identificação do artigo, tipificação metodológica e caracterização da amostra. Foram ainda extraídos os fragmentos dos textos que melhor respondessem às variáveis de descrição do conteúdo, com tradução livre dos trechos pelas autoras. A busca de artigos e a análise dos resultados foram realizadas em maio de 2018.

## RESULTADOS

Foram identificados 212 artigos. Desses, inicialmente foram excluídos sete estudos por não estarem no período selecionado e, posteriormente, foram excluídos 63 textos repetidos. Após a leitura de títulos e resumos, excluíram-se, respectivamente, 85 e 36 estudos que discorriam sobre temáticas diferentes do objeto desta casuística, restando 21 artigos para leitura na íntegra.

**FIGURA 1:**
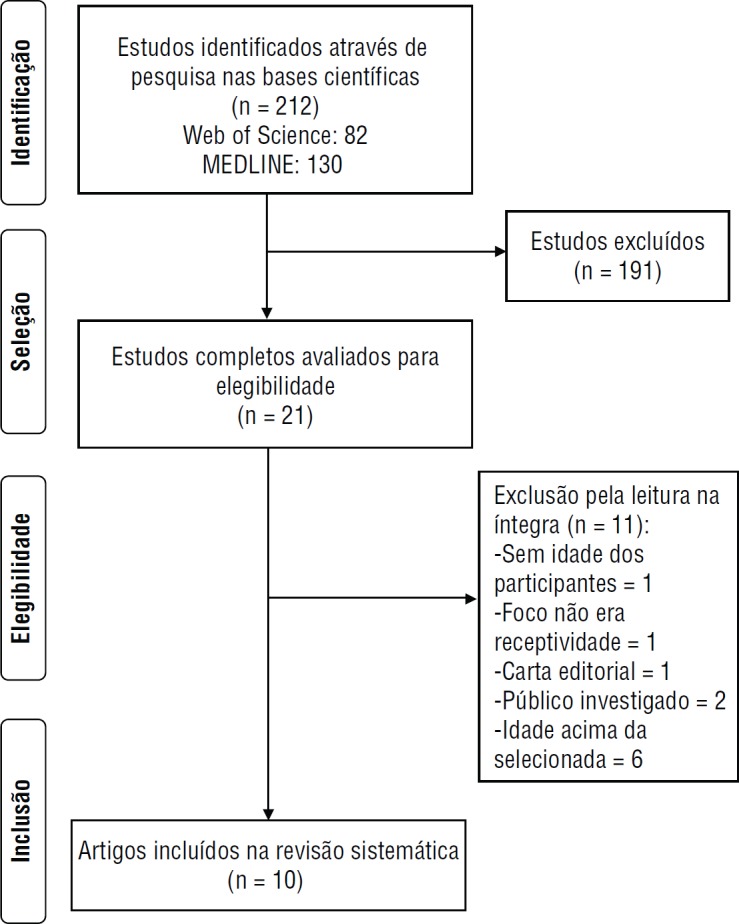
Fluxograma de seleção dos artigos sobre receptividade à vacina contra o papilomavírus humano

Dos 21 artigos selecionados a partir da leitura integral, 11 foram excluídos: um não relatava a idade dos participantes; em outro, o foco não era a receptividade; outro era uma carta ao editor; em dois o público investigado era formado exclusivamente pelos responsáveis; e seis apresentavam parte de suas amostras com idade muito acima da selecionada para esta revisão. Ao final, 10 artigos foram selecionados, como mostra a figura 1.

A tabela 1 apresenta a caracterização dos estudos selecionados (14, 16, 18, 27-33). A maior parte dos estudos não apresentou conceitos de aceitação e/ou adesão, havendo somente dois artigos (31, 32) que reportaram definições. Outras informações alusivas ao grau de receptividade e suas formas de avaliação, ao tipo de receptividade reportada e aos conceitos adotados pelos autores aparecem na tabela 2.

**TABELA 1:** Estudos sobre receptividade à vacina contra o papilomavírus humano publicados entre 2006 e 2017

Autor/Ano (Referência)	País	Desenho	NE^a^	Técnica de coleta de dados	Análise estatística	Sujeito^b^	Amostra (n)
Gottvall et al., 2009 (14)	Suécia	Transversal	2c	Questionário autoaplicado	Descritiva^c^, teste t e qui-quadrado	Adolescentes (M e F não vacinados) 14-19 anos	608
Rand et al., 2011 (27)	Estados Unidos	Transversal	2c	Entrevista estruturada via telefone	Descritiva^c^, regressão logística e qui-quadrado	Adolescentes (M e F vacinados e não vacinados) 15-17 anos e responsáveis	208
Kilic et al., 2012 (28)	Turquia	Transversal	2c	Questionário autoaplicado	Descritiva^c^, regressão logística e teste de concordância de Kappa	Adolescentes (F não vacinadas) 17-22 anos e responsáveis	301
Gutierrez et al., 2013 (29)	Estados Unidos	Transversal (quali-quantitativo)	2c	Questionário autoaplicado com escala Likert; entrevista - grupo focal	Descritiva^c^	Adolescentes (M não vacinados) 13-21 anos	86
Poole et al., 2013 (30)	Mali	Transversal	2c	Entrevista estruturada presencial	Descritiva^c^, teste de McNemar e exato de Fisher	Adolescentes (M e F não vacinados) 12-17 anos e responsáveis	25
Gellenoncourt e Patrizio, 2014 (16)	França	Transversal	2c	Questionário autoaplicado	Descritiva^c^, teste qui-quadrado	Adolescentes (M não vacinados) 14-21 anos	326
Turiho et al., 2014 (31)	Uganda	Transversal (quali-quantitativo)	2c	Questionário autoaplicado com escala Likert	Descritiva^c^, regressão logística, qui-quadrado de Pearson e análise temática	Crianças e adolescentes (F vacinados e não vacinados) 9-19 anos	777
Khurana et al., 2015 (32)	Estados Unidos	Transversal	2c	Questionário autoaplicado	Descritiva^c^, análise de variância, regressão logística e qui-quadrado de Pearson	Adolescentes (M não vacinados) 11-21 anos e responsáveis	154
Botha et al., 2015 (33)	África do Sul	Coorte	2b	Entrevista estruturada presencial	Descritiva^c^	Crianças e adolescentes (F vacinadas e não vacinadas) > 9 anos	2 030
Rahman et al., 2017 (18)	Estados Unidos	Transversal	2c	Questionário *National Immunization Survey-Teen* via telefone e entrevista domiciliar	Descritiva^c^, regressão logística e qui-quadrado	Adolescentes (F vacinadas e não vacinadas) 13-17 anos	9 403

a NE = nível de evidência.

b Sujeitos: avaliados quanto ao sexo (M: masculino; F: feminino), idade, estado vacinal.

c Estatística descritiva: frequência absoluta e relativa, média e/ou desvio padrão.

**TABELA 2:** Receptividade reportada, conceitos de aceitação e/ou adesão, formas de avaliação e grau de receptividade em artigos relativos à vacina contra o papilomavírus humano, 2006 a 2017

Autor/Ano (Referência)	Receptividade	Conceito	Forma de avaliação	Grau de receptividade
Gottvall et al., 2009 (14)	Aceitação/adesão	Ausente	Pergunta: Você já se vacinou contra o HPV? Se não se vacinou, você gostaria de se vacinar?	Aceitação de 84%; 65% dos alunos não sabiam se tinham recebido a vacina, porém 84% gostariam de se vacinar.
Rand et al., 2011 (27)	Aceitação	Ausente	Pergunta: Você aceitou ou recusou a vcHPV? Caso seus pais quisessem, você se vacinaria? Fórmula: Taxa de aceitação/adoção: percentual de aceites entre adolescentes Taxa de recusa: percentual de recusas entre adolescentes	Aceitação de 75%, sendo que 16% dos adolescentes mais velhos recusaram a vacina; mais de 50% das adolescentes já haviam recebido uma dose da vacina com menos de 25% de recusa.
Kilic et al., 2012 (28)	Aceitação	Ausente	Pergunta: Você gostaria de se vacinar contra o HPV?	43,5% das meninas aceitariam se vacinar, 37,2% estavam indecisas e 19,3% não desejariam se vacinar.
Gutierrez et al., 2013 (29)	Aceitação	Ausente	Pergunta: Qual a probabilidade de você se vacinar contra o HPV no próximo ano?	Os participantes tiveram atitudes moderadas a favoráveis ​​em relação à vacinação.
Poole et al., 2013 (30)	Aceitação	Ausente	Pergunta: Você gostaria que a vcHPV estivesse disponível no país? Caso estivesse disponível, você receberia esta vacina?	100% dos participantes aceitariam a vacina caso estivesse disponível.
Gellenoncourt e Patrizio, 2014 (16)	Aceitação	Ausente	Pergunta: Você aceitaria a vcHPV se ela estivesse disponível para meninos na França?	40% dos participantes estavam indecisos, 33% aceitariam a vacinação, 23% rejeitariam e 4% não responderam.
Turiho et al., 2014 (31)	Aceitação/ Adesão	Vontade/resistência em receber a vacina e em completar as três doses	Pergunta: Você aconselharia seus amigos a se vacinarem contra o HPV? Você permitiria que sua filha se vacinasse no futuro?	Aceitação em 91% da amostra, dos quais 56% eram vacinados e 43% não vacinados.
Khurana et al., 2015 (32)	Aceitação	Aceitação: querer a vacina; aceitação condicional: querer a vacina caso proteja contra verrugas/ câncer cervical	Pergunta: Você aceita receber a vcHPV? Você aceita receber a vacina caso ela proteja contra verrugas/câncer cervical?	Aceitação em 16% na amostra; a maioria dos participantes era indeciso; após a informação de que a vacina protegeria contra verrugas/câncer cervical, a aceitação condicional a essa proteção foi de 61%.
Botha et al., 2015 (33)	Adesão	Ausente	Fórmula: Taxa de adesão: percentual de meninas vacinadas entre convidadas Taxa de conclusão: percentual de meninas que completaram as doses entre vacinadas	Adesão em 91,6% da amostra, dos quais 87,8% receberam três doses e 3,8% receberam duas doses.
Rahman et al., 2017 (18)	Adesão	Ausente	Fórmula: Taxa de iniciação: percentual de meninas que iniciaram a imunização entre participantes Taxa de completude: percentual de meninas que completaram as doses entre participantes	57,3% das adolescentes iniciaram o esquema vacinal, porém somente 39,1% completaram as três doses.

Em geral, os estudos relataram uma favorável, porém heterogênea, receptividade à vcHPV, com aceitação oscilando de 16% a 100% dos sujeitos em sete artigos (14, 16, 27, 28, 30-32) e adesão variando de 39% a 91% dos participantes em três artigos (18, 31, 33), havendo ainda um estudo que investigou a aceitação de forma qualitativa com termos “moderada a favorável” (29). Foram identificados 11 facilitadores de alta receptividade (16, 27-33) e nove barreiras alusivas à baixa aceitação e adesão (14, 16, 27-31), descritos na tabela 3.

## DISCUSSÃO

A existência de artigos sobre o tema indica que a receptividade à vcHPV é uma preocupação coletiva em diversos países, o que pode estar associado à distribuição global do vírus (1-2, 4-5, 12) e ao elevado potencial desse imunobiológico para reduzir a incidência do câncer cervical (34). Essas questões justificam o interesse internacional em realizar estudos para compreender com maior clareza a aplicabilidade da vacina, bem como as barreiras e os facilitadores à receptividade pelo público alvo nas diversas regiões estudadas (10, 35-39).

Alguns estudos aprofundaram outros tópicos relacionados à receptividade, como a ocorrência de eventos adversos (29, 31) e a compreensão quanto ao risco de infecção (14, 28). Isso mostra que a receptividade é uma questão transversal, que envolve múltiplas temáticas, desde a saúde da criança, perpassando pelo adolescente, a mulher e o homem, até a promoção e a proteção da saúde reprodutiva, a prevenção de infecções sexualmente transmissíveis e as políticas públicas (7, 10).

Quanto à qualidade da evidência, o predomínio de artigos com nível de evidência 2c pressupõe estudos com delineamento metodológico adequado e razoável credibilidade (25). Ademais, menciona-se que pesquisas focadas em “receptividade” são realizadas a partir de delineamentos transversais ou de coorte, o que decorre do fato de a temática ser melhor mensurada por esses desenhos, que permitem a análise do fenômeno conforme a hipótese levantada pelo pesquisador (40). Todavia, estudos observacionais quantitativos permitem uma análise primária (41) descritiva do fenômeno para que, posteriormente, o mesmo seja avaliado em profundidade pelos sujeitos que o vivenciam numa perspectiva qualitativa (29).

**TABELA 3:** Facilitadores e barreiras da receptividade à vacina contra o papilomavírus humano nas publicações de 2006 a 2017

Fator	Citado em
**Facilitador**	
1. Conhecimento referente à vacina/HPV	29, 31, 32
2. Desejo de prevenção	27, 28
3. Experiência de amigos	31, 32
4. História de atividade sexual/promiscuidade	29, 32
5. Necessidade/presença de recomendação médica	27, 28
6. Boa percepção do risco de infecção	16, 27
7. Ocorrência mínima de reações adversas	31
8. Disponibilidade de vacina gratuita	30
9. Renda anual > US$ 75.000	32
10. Finalização das doses no ano letivo	33
11. Comunicação efetiva	33
**Barreira**	
1. Falta/inadequada informação relativa à vacina	16, 27, 28, 31
2. Ausência/baixa percepção do risco de infecção	14, 16, 27, 28
3. Ocorrência de reações adversas	29, 31
4. Pouca autonomia de decisão/não autorização dos pais	14, 30
5. Desconfiança/medo da vacina/agulha	14, 16
6. Alto custo	14
7. Dor	14
8. Falta de recomendação médica	16
9. Ausência de desejo	27

Dentro dessa vertente, a utilização de abordagem quali-quantitativa ocorre pela existência de dados que são mensurados de modo mais preciso com questionários quantitativos e informações que necessitam de técnicas qualitativas para um melhor entendimento (42). Essa junção tem a finalidade de explorar melhor a temática, pois ambos os enfoques são complementares (37, 42), refletindo o caráter multifacetado do fenômeno receptividade, cuja compreensão parece exigir diferentes abordagens (37). Evidenciou-se tal situação em um estudo no qual, embora a metade dos participantes relatasse ter entendimento acerca da vacina, o debate em grupo focal detectou entendimento incompleto (29).

Ao avaliar o público-alvo, a maioria dos autores preferiu investigar a perspectiva feminina, viés provavelmente vinculado ao fato de o câncer cervical acometer apenas mulheres (5) e em virtude de os meninos ainda não serem prioritários em alguns programas de imunização (2, 7). Contudo, gradativamente, as campanhas nacionais estão abarcando o sexo masculino (3, 16). Essa mudança provavelmente será refletida em pesquisas futuras e trará benefícios em razão de os meninos também serem suscetíveis ao HPV (17). Ademais, os meninos exercem um papel fundamental na transmissão do vírus, constituindo um público carente de maior atenção (6), visto iniciarem a vida sexual de forma mais precoce e ativa do que as meninas, podendo apresentar bons resultados em programas de vacinação (2, 27).

Acredita-se que a existência de estudos que também investigam a perspectiva dos responsáveis ocorra em virtude da limitação na liberdade de escolha (2, 10, 27, 30) dos adolescentes em relação aos pais (28, 34), pois a decisão de vacinar diverge conforme a autonomia do sujeito (30). Ademais, existem pais que se responsabilizam apenas pela imunização preconizada à infância e não se sentem responsáveis pela imunização dos adolescentes, como também pais que não autorizam a administração da vcHPV aos adolescentes (2, 14, 36) por receio de a mesma resultar em estímulo ao ato sexual (3, 39). Nesses estudos, é desafiador interpretar as razões pelas quais um adolescente não se vacinou, pois nem sempre está claro se o participante relatou a não adesão devido à própria visão ou à visão dos pais (36), sendo algumas vezes necessário que o adolescente seja investigado sem a presença do responsável.

Apesar de existirem instrumentos validados (17) que mensuram os graus de receptividade e seus determinantes, não foi possível identificar nos estudos um padrão ouro relativo à avaliação o qual fosse baseado em conceitos precisos de aceitação e adesão. Assim, observou-se apenas uma tendência quanto ao uso de questionários autoaplicados os quais investigam diretamente o público-alvo.

O fato de a maior parte dos autores utilizarem questionários autoaplicados é explicado pela maior privacidade que a técnica permite aos participantes diante de um tema delicado como a sexualidade (36-39). Além disso, os questionários autoaplicados envolvem maior praticidade, permitem a avaliação de uma amostra maior e já tiveram sua sensibilidade demonstrada para mensurar a receptividade a partir de análise quantitativa (quando é avaliado o percentual de vacinados) (14, 16, 18, 27, 28, 30-33) e qualitativa (usando termos como “baixa, moderada e alta” receptividade) (29).

### Conceitos e fatores relacionados à receptividade

Nesta revisão, não foi observada uma padronização relativa aos conceitos de aceitação e adesão: os autores não apontaram definições de aceitação e foram apresentados apenas dois conceitos incompletos de adesão. Apesar das semelhanças, “aceitação” diverge de “adesão”, pois a primeira geralmente precede a segunda, mesmo na hipótese de pessoas aceitarem a vacina como uma boa intervenção sem que de fato se vacinem (17). A adesão não seria questionar se o indivíduo deseja receber a vacina ou aceita o imunobiológico, mas, isso sim, confirmar que a pessoa foi vacinada, preferencialmente com conferência do cartão vacinal.

A partir dos estudos analisados (31, 32, 39), elaborou-se um conceito de aceitação: intenção voluntária de receber uma vacina ou concordar que a mesma representa uma boa estratégia preventiva. De modo similar, sistematizou-se o conceito de adesão (31, 39, 43, 44): iniciar a vacinação e completar o esquema proposto, considerando o número de doses recomendadas e o intervalo entre as mesmas.

A receptividade favorável contribui para a principal prioridade dos programas de imunização: a obtenção de altas coberturas vacinais (15). Contudo, o grau de receptividade e os fatores relacionados oscilam entre os países, variações que podem estar associadas a aspectos culturais (2, 3), além de estarem relacionadas à forma como os serviços de saúde são organizados.

O fato de a aceitação à vacina apresentar-se maior que a adesão é preocupante, pois não basta as pessoas serem conscientes quanto à relevância da vacinação – é preciso que se vacinem de fato e que a conscientização esteja alinhada ao ato vacinal. A não adesão – embora possa não repercutir em malefícios ­diretos aos indivíduos – pode dificultar o adequado controle do câncer cervical e produzir um retardo na queda de indicadores de morbidade e mortalidade relacionados ao HPV em consequência da vacina não demonstrar todo o seu potencial (1, 5, 34). Assim, é preciso analisar os preditores que influenciam a receptividade para compreender a lacuna entre uma aceitação elevada e uma adesão desproporcional.

Conforme evidenciado neste estudo, o conhecimento relativo ao tema pode atuar como barreira ou facilitador à receptividade. Assim, os conhecimentos gerados pela ciência podem ser empregados para educar pessoas e aumentar a receptividade, como também a carência de dados seguros ou as informações distorcidas podem gerar medo e elevar a recusa. Observa-se a segunda situação em contextos nos quais a veiculação de dados sem evidências científicas por redes sociais ou por grupos contrários à vacinação (34, 37) geram repercussões negativas permanentes e de difícil reversão, causando dificuldade de receptividade às vacinas (45).

Constata-se uma tendência nos estudos de receptividade de avaliar o nível de conhecimento dos participantes em relação à vacina e ao HPV, pressupondo que um maior grau de informação sobre o tema aumentaria a aceitabilidade entre adolescentes (14, 32). Contudo, alguns estudos não mostram essa relação: um estudo apontou baixo conhecimento na maioria das populações com alta aceitação (14); e outro estudo evidenciou um nível aparentemente satisfatório de informação entre estudantes, todavia com coberturas vacinais presumivelmente insuficientes (35). Nessa vertente, estudiosos apontam que o conhecimento não predispõe à aceitação da vacina, pois outros elementos teriam maior nexo que o conhecimento em si (17, 31).

Dentre os fatores de destaque relativos à alta receptividade, aponta-se o desejo de prevenção, que evidencia um ponto positivo associado aos cuidados em saúde, visto estar relacionado a mudanças de estilo de vida e redução dos fatores de risco (46). De modo contrário, a baixa percepção quanto ao risco de infecção é preocupante, já que pode estar vinculada à exposição a situações de risco e gera pouco envolvimento com a imunização (47). Assim, um ponto comum entre o facilitador “desejo de prevenção” e a barreira “baixa percepção” é a dependência de ambos do padrão de comportamento do sujeito frente ao problema.

Verifica-se também que o fator econômico é determinante à receptividade. O preço elevado do imunobiológico não consistiu em impedimento para aqueles com renda anual acima de US$ 75 000 (32), e, de forma contrária, apresentou-se como obstáculo à imunização para pessoas de baixa renda (14). Isso ocorre, em especial, nos países sem cobertura universal de saúde (20), diferentemente de locais onde a vacinação gratuita está associada a uma alta adesão (37). Nessa conjuntura, o padrão de renda é considerado um determinante social que define a situação de saúde (46), por estar relacionado ao acesso ao imunobiológico e a uma melhor condição de saúde devido aos benefícios da vacina.

Além dos fatores aqui salientados, é preciso considerar aqueles pontuados em outros estudos e que se relacionam à aceitação (8): a capacitação dos profissionais de saúde (20) e o modo como se caracterizam as crenças e as atitudes dos pais, das crianças e dos adolescentes frente à imunização (3). Assim, acredita-se que intervenções direcionadas a esses preditores possam motivar uma maior adesão.

Embora a vcHPV já seja uma intervenção altamente recomendada (10), ainda existem barreiras à sua adoção que podem resultar em baixas taxas de vacinação (17, 38, 39) e em coberturas vacinais menores que as apresentadas por outros imunobiológicos (39). Os motivos da recusa são numerosos e complexos (39), sendo as barreiras aqui reportadas relatos comuns em estudos semelhantes (34-37). Todavia, há outro preditor alusivo à baixa receptividade não evidenciado pelos estudos desta revisão, que seria a pouca evidência em relação à eficácia (23) e à segurança do imunobiológico (36-38). Esse preditor merece atenção, pois causa receio e faz as pessoas postergarem ou recusarem a imunização (39).

Ao estudar o contexto da imunização, alguns autores verificaram equívocos associados a sintomas supostamente relacionadas à vacina (39), os quais geram medo e tabus (34, 37). Embora os eventos adversos sejam geralmente breves e incomuns (36), a preocupação com tais reações é considerada um impedimento à imunização (34), sendo possivelmente uma causa de baixa adesão (3, 7, 8, 31). Sendo assim, sugere-se a abordagem do tema com atividades educativas (2) antes da vacinação.

Apesar da limitação de informações devido aos poucos artigos selecionados, considera-se que os dados supriram parte de uma lacuna existente. Aponta-se como limitação o fato de os artigos analisados apresentarem pouca interface com a abordagem qualitativa e a literatura cinzenta, não contemplarem estudos da América Latina, terem predominância de delineamento transversal e insuficiente análise da receptividade na visão das crianças. Tais limites não invalidam os resultados evidenciados, porém levantam a hipótese da existência de um universo ainda desconhecido a ser explorado.

Após a análise dos artigos selecionados, foi possível caracterizar a receptividade à vcHPV, sendo observada uma receptividade favorável mas heterogênea, identificada mais por aceitação do que por adesão. Ademais, foram descritos os fatores relacionados à receptividade, aqui especificados como barreiras ou facilitadores.

A análise da receptividade não finaliza aqui. Sugere-se a realização de novos estudos que verifiquem como a informação alusiva à vacina e ao HPV tem sido compreendida e associada ao grau de receptividade; que esclareçam como cada preditor contribui à receptividade; e que construam instrumentos para mensurar a aceitação e a adesão de modo fundamentado em definições precisas para compreender melhor o fenômeno.

Os achados evidenciados são de interesse dos programas nacionais de imunização, tendo esta casuística a intenção de facilitar o acesso ao tema por profissionais de saúde. Espera-se que este artigo contribua para a consolidação de políticas públicas e estratégias educativas focadas no estímulo à vacinação, de modo a aumentar a receptividade e promover coberturas vacinais adequadas.

## Contribuição das autoras.

LELS concebeu e desenhou a pesquisa, realizou a busca dos dados e redigiu o artigo. Todas as autoras (LELS, MLCO, DG) analisaram e interpretaram os dados e revisaram criticamente o conteúdo, além de revisarem e aprovarem a versão final.

## Agradecimentos.

Aos professores e alunos do Programa de Pós-Graduação em Ciências e Tecnologia da Saúde da Universidade de Brasília (PPGCTS/UnB) e aos membros do grupo de pesquisa Acesso a Medicamentos e Uso Racional (AMUR) pelo incentivo na realização deste estudo; à Coordenação de Aperfeiçoamento de Pessoal de Nível Superior (CAPES) por apoiar a pesquisa científica no Brasil; aos colegas da Procuradoria Regional do Trabalho da 10º Região, vinculado ao Ministério Público do Trabalho (MPT/MPU), pela parceria de sempre.

## Conflitos de interesse.

Nada declarado pelas autoras.
